# Characteristics and determinants of pulmonary long COVID

**DOI:** 10.1172/jci.insight.177518

**Published:** 2024-04-23

**Authors:** Michael John Patton, Donald Benson, Sarah W. Robison, Dhaval Raval, Morgan L. Locy, Kinner Patel, Scott Grumley, Emily B. Levitan, Peter Morris, Matthew Might, Amit Gaggar, Nathaniel Erdmann

**Affiliations:** 1 Medical Scientist Training Program, Heersink School of Medicine,; 2 Hugh Kaul Precision Medicine Institute,; 3 Department of Radiology,; 4 Division of Pulmonary, Allergy, and Critical Care Medicine, Department of Medicine, and; 5 Department of Epidemiology, University of Alabama at Birmingham, Birmingham, Alabama, USA.; 6 Birmingham VA Medical Center, Pulmonary Section, Birmingham, Alabama, USA.; 7 Department of Medicine, Division of Infectious Diseases, University of Alabama at Birmingham, Birmingham, Alabama, USA.

**Keywords:** Infectious disease, Pulmonology, Bioinformatics, Diagnostic imaging, Diagnostics

## Abstract

**BACKGROUND:**

Persistent cough and dyspnea are prominent features of postacute sequelae of SARS-CoV-2 (also termed “long COVID”); however, physiologic measures and clinical features associated with these pulmonary symptoms remain poorly defined. Using longitudinal pulmonary function testing (PFT) and CT imaging, this study aimed to identify the characteristics and determinants of pulmonary long COVID.

**METHODS:**

This single-center retrospective study included 1,097 patients with clinically defined long COVID characterized by persistent pulmonary symptoms (dyspnea, cough, and chest discomfort) lasting for 1 or more months after resolution of primary COVID infection.

**RESULTS:**

After exclusion, a total of 929 patients with post-COVID pulmonary symptoms and PFTs were stratified as diffusion impairment and pulmonary restriction, as measured by percentage predicted diffusion capacity for carbon monoxide (DLCO) and total lung capacity (TLC). Longitudinal evaluation revealed diffusion impairment (DLCO ≤ 80%) and pulmonary restriction (TLC ≤ 80%) in 51% of the cohort overall (*n* = 479). In multivariable modeling regression analysis, invasive mechanical ventilation during primary infection conferred the greatest increased odds of developing pulmonary long COVID with diffusion impairment and restriction (adjusted odds ratio [aOR] = 9.89, 95% CI 3.62–26.9]). Finally, a subanalysis of CT imaging identified radiographic evidence of fibrosis in this patient population.

**CONCLUSION:**

Longitudinal PFTs revealed persistent diffusion-impaired restriction as a key feature of pulmonary long COVID. These results emphasize the importance of incorporating PFTs into routine clinical practice for evaluation of long COVID patients with prolonged pulmonary symptoms. Subsequent clinical trials should leverage combined symptomatic and quantitative PFT measurements for more targeted enrollment of pulmonary long COVID patients.

**FUNDING:**

National Institute of Allergy and Infectious Diseases (AI156898, K08AI129705), National Heart, Lung, and Blood Institute (HL153113, OTA21-015E, HL149944), and the COVID-19 Urgent Research Response Fund at the University of Alabama at Birmingham.

## Abstract 2

Pulmonary function testing should be integrated into routine diagnostic practice to evaluate patients with prolonged pulmonary symptoms after resolution of SARS-CoV-2 infection.

## Graphical abstract

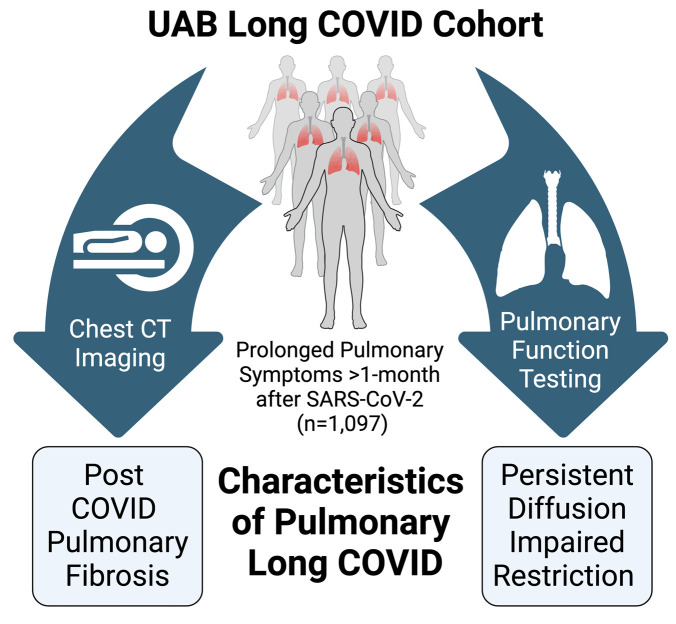

## Introduction

A major consequence of the COVID-19 pandemic has been the complex and frequently debilitating postacute sequelae of SARS-CoV-2 infection (PASC, also termed “long COVID”), estimated to occur in 10% of patients after primary infection (1–2). To date, over 150 distinct long COVID symptoms involving every major organ system have been reported (3). Recent efforts to develop a consensus definition for long COVID using symptom clustering have highlighted broad disease sub-types associated with chronic fatigue, postexertional malaise, brain fog, and loss of smell or taste (4). These efforts represent an important first step for long COVID research; however, characterizing discreet endotypes with widely available quantitative physiologic measurements is essential for standardizing long COVID diagnosis and management.

Prolonged pulmonary symptoms, notably dyspnea and cough, are among the most commonly reported manifestations of long COVID (hereon referred to as “pulmonary long COVID”) (4–6). Although pulmonary complaints are frequently reported, the underlying cause(s) and clinical trajectory of patients suffering these symptoms remains unclear. Prior studies have suggested diffusion impairment that resolves within 1 year of hospitalization is a common feature of postacute COVID-19 (7–10). Other postacute COVID follow-up studies have reported radiologic evidence of lung pathology characterized by ground glass opacities and fibrotic changes (11–18). While these results have provided valuable insight into post-COVID lung pathology, these studies are limited by the cohort size and inherently biased toward postacute COVID patients, rather than long COVID populations experiencing persistent pulmonary complaints.

To address this knowledge gap, we leveraged a large demographically diverse cohort exclusively composed of long COVID patients experiencing pulmonary symptoms for 1 or more months after resolution of SARS-CoV-2 infection. Using longitudinal pulmonary function testing (PFT) and computerized tomography (CT) imaging, we identify specific disease features and risk factors linked to the development of pulmonary long COVID. These presentations capture the population of patients with persistent clinical symptoms and reflect the natural history of pulmonary long COVID. Lastly, we provide evidence for a previously undescribed endotype of long COVID defined by persistent diffusion impairment and pulmonary restriction.

## Results

### Study population.

**Figure 1:**
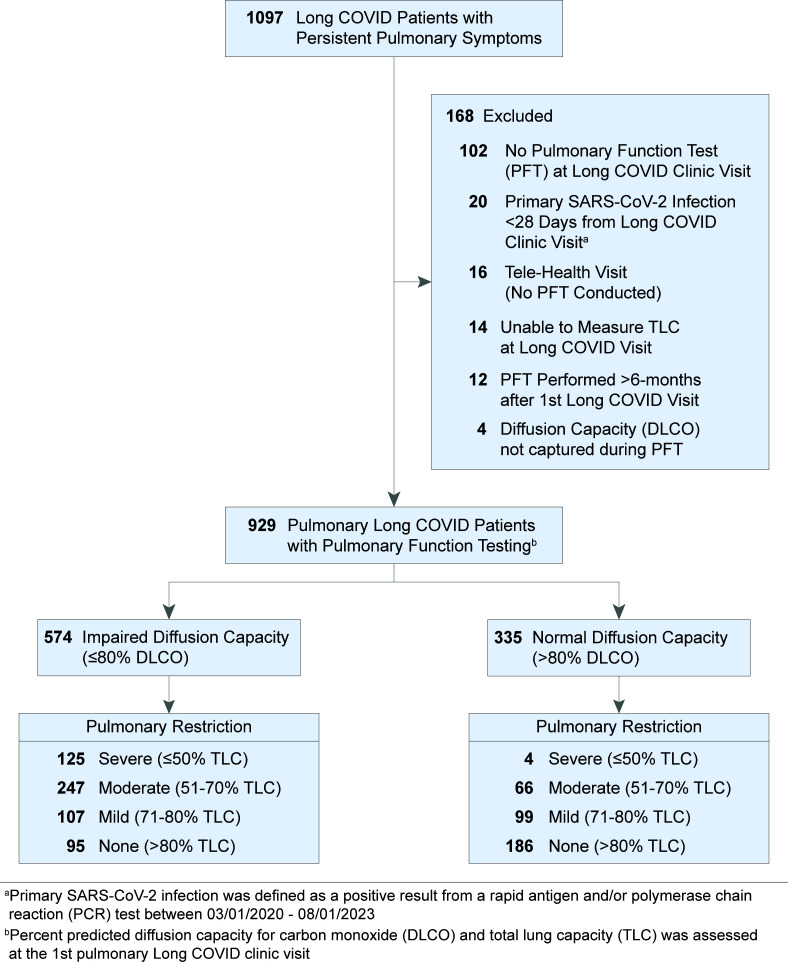
Accrual of long COVID patients with persistent pulmonary symptoms in the UAB Health System. Study flow chart.

A total of 1,097 patients with prolonged pulmonary symptoms after primary SARS-CoV-2 infection were identified based on their evaluation in a postacute COVID clinic. After exclusion, 929 patients with prolonged pulmonary symptoms (dyspnea, cough, or chest discomfort) lasting 1 or more months after resolution of primary SARS-CoV-2 infection and a subsequent PFT were included in this study (Figure 1 and Tables 1 and 2). To evaluate the role of pulmonary abnormalities in diffusion capacity for carbon monoxide (DLCO) and evidence of profibrotic processes, we stratified patients by diffusion impairment and severity of pulmonary restriction as measured by first postacute PFT. Median time from primary SARS-CoV-2 infection to first long COVID clinic visit was 125 days for diffusion-impaired patients and 148 days for patients with normal diffusion capacity (Tables 1 and 2). Patient age (mean ± SD) for the diffusion-impaired group was 56 ± 13 years compared with 48 ± 14 years in the normal diffusion group (Tables 1 and 2). For both groups, the majority of primary SARS-CoV-2 infections occurred during the Alpha-variant wave (range: 62%–63%), followed by 21%–23% and 15%–16% occurring in the Delta and Omicron waves, respectively (Supplemental Table 2; supplemental material available online with this article; https://doi.org/10.1172/jci.insight.177518DS1). Differences in primary pulmonary symptom reported at first long COVID clinic visit were unremarkable between patients with and without diffusion impairment (range: dyspnea, 76%–79%; cough, 17%–19%; chest discomfort, 4%).

We sought to determine how acute disease severity contributed to PFT findings for patients with pulmonary long COVID. Broadly, requiring higher-intensity respiratory support was associated with diffusion impairment and restriction. Acute disease severity was stratified by peak World Health Organization (WHO) ordinal score. Of the 73 patients who required invasive mechanical ventilation (IMV), only 3 were in the normal diffusion capacity group (Tables 1 and 2). Increased frequency and duration of oxygen support was observed among diffusion-impaired patients in the severe restriction group (IMV: 32%, high-flow nasal cannula [HFNC]: 22%), exceeding that of the moderate (IMV: 9%, HFNC: 16%), mild (IMV: 6%, HFNC: 14%), and no-restriction groups (IMV: 3%, HFNC: 5%; Tables 1 and 2). A similar trend of increased time of oxygen therapy (median and IQR [Q1–Q3]) during primary infection was also observed in diffusion-impaired patients (IMV: 18 [IQR 8–35] days, HFNC: 5 [IQR 3–8] days, and nasal cannula: 7 [IQR 4–11] days; Supplemental Table 2). Differences in vaccination, preexisting pulmonary disease or obstructive sleep apnea, and smoking history prior to primary SARS-CoV-2 infection were minimal between diffusion-impaired and normal groups (Tables 1 and 2). Patients with diffusion impairment had a greater proportion of preexisting diabetes (21%) and heart failure or hypertension (52%) compared with the normal diffusion group, with 14% and 33%, respectively (Tables 1 and 2). Cumulative lung involvement (25 points total, 5 points per lobe; median and IQR [Q1–Q3]) was higher in the diffusion-impaired group (9 [IQR 1–17]) compared with the normal group (0 [IQR 0–2]). The degree of lung involvement on CT imaging for diffusion-impaired patients correlated with increasing severity of lung restriction (none: 3 [IQR 1–7], mild: 3 [IQR 0–9], moderate: 7 [IQR 1–13], severe: 17 [IQR 9–21]; Tables 1 and 2). Further characteristics of the cohort are presented in Tables 1 and 2 and Supplemental Table 2.

### Longitudinal evaluation of PFT.

**Figure 2:**
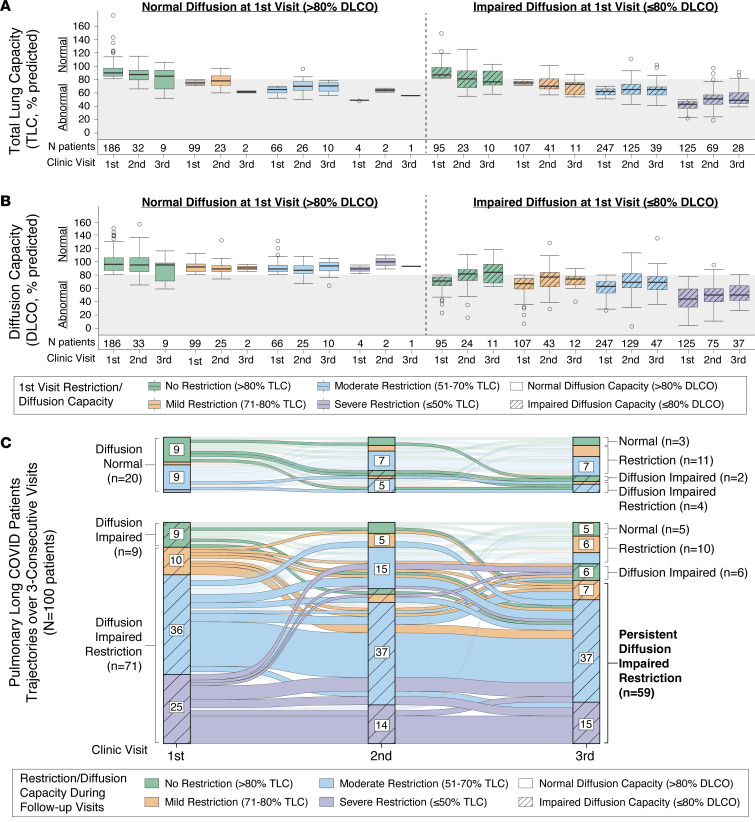
Diffusion-impaired restriction is a key feature of persistent pulmonary long COVID. (**A** and **B**) Results of percentage predicted total lung capacity (TLC) (**A**) and diffusing capacity for carbon monoxide (DLCO) (**B**) are shown for 3 clinic visits with stratification by restriction severity (color) measured during the first visit PFT and presence or absence of diffusion impairment (hashed lines). Box-and-whisker plots represent the median (black center line) and 25th and 75th percentiles (box boundaries) for each PFT measured, with number of patients (*n*) reported below each group. Normal TLC and DLCO are denoted by the gray color on the plot at 80%. (**C**) Alluvial diagram displays patient PFT trajectories over 3 consecutive visits (*n* = 100 total) with relative improvement or decline as measured by TLC (color) and DLCO (hashed lines). Labeling of alluvial diagram axes groups with fewer than 5 patients was omitted for visual clarity.

To assess physiological differences between patients with pulmonary long COVID, we evaluated longitudinal PFTs over 3 total long COVID visits. After first-visit stratification, patients without diffusion impairment had, on average, normal lung capacity during the second visits (total lung capacity [TLC] first: 82% ± 15%, second: 79% ± 13%), with mild decline on third visit (72% ± 15%) compared with patients with diffusion impairment on respective follow-up visits (TLC first: 6%5 ± 18%, second: 65% ± 15%, third: 64% ± 15%) (Figure 2A and Supplemental Table 4). Diffusion-impaired patients with severe or moderate restriction experienced little to no improvement in TLC at second (severe: 51% ± 14%, moderate: 66% ± 11%) or third visit (severe: 55% ± 14%, moderate: 65% ± 12%) (Figure 2A and Supplemental Table 4). Overall, patients with normal diffusion capacity at first visit maintained normal or above normal DLCO at the follow-up visits (DLCO first: 95% ± 12%, second: 93% ± 16%, third: 90% ± 15%), regardless of the level of first-visit restriction (Figure 2B, Supplemental Table 4). In contrast, patients with diffusion impairment at first visit remained, on average, diffusion impaired at all follow-up visits (DLCO first: 59% ± 16%, second: 66% ± 19%, third: 65% ± 19%), with a clear association between worsening TLC and DLCO (Figure 2B and Supplemental Table 4). Additional PFT measurements are provided in Supplemental Table 4.

Alluvial diagrams were used to assess improvement or decline in diffusion impairment and restriction for patients with 3 consecutive PFTs at the University of Alabama at Birmingham (UAB) Post-COVID Pulmonary Clinic (*n* = 100, Figure 2C). Overall, we observed that the majority of patients with diffusion impairment and severe or moderate restriction at first visit had persistent restriction and diffusion impairment at second and third follow-up visits (Figure 2C). Improvement from diffusion impairment at any level of restriction was rare, with only 5 patients regaining normal TLC and DLCO by their third visit. Over half of the normal diffusion capacity patients (*n* = 11) had some level of lung restriction at first visit (*n* = 1 mild, *n* = 9 moderate, *n* = 1 severe) as well as their third visit (*n* = 11 restricted). Notably, 7 of the 9 patients with normal TLC and DLCO at first visit had some level of restriction and/or diffusion impairment by the third visit (Figure 2C). This observation among patients with normal lung function appears to represent an ongoing and progressive pulmonary process resulting in restriction and/or diffusion impairment. Overall, restriction or diffusion-impaired restriction were the predominant phenotypes observed by the third follow-up visit, thereby indicating an earlier stage of disease followed by progression among the patients with normal diffusion capacity (Figure 2C).

### Risk factors for developing pulmonary long COVID with severe pulmonary restriction.

Logistic regression models were used to identify risk factors for developing long COVID with combined diffusion impairment (DLCO ≤ 80%) and severe or moderate restriction (TLC ≤ 70%). Unadjusted univariable modeling revealed (median and 95% CI]) advanced age of 65 years or older (OR = 1.63 [95% CI 1.23–2.23]), male sex (OR = 1.88 [95% CI 1.43–2.48]), renal disease (OR = 2.48 [95% CI 1.47–4.15]), diabetes (OR = 1.82 [95% CI 1.30–2.51]), heart failure or hypertension (OR = 2.54 [95% CI 1.98–3.32]), smoking history (OR = 1.39 [95% CI 1.03–1.86]), ICU admission (OR = 6.48 [95% CI 4.45–10.0]), and use of nasal cannula (OR = 4.21 [95% CI 2.90–6.07]), HFNC (OR = 6.10 [95% CI 3.92–9.82]), and IMV (OR = 16.0 [95% CI 8.87–34.4]) as independent risk factors for developing pulmonary long COVID with severe or moderate restriction (Table 3). After adjusting for all variables, we observed that IMV conferred the greatest increased odds of developing pulmonary long COVID with diffusion impairment and severe or moderate restriction (adjusted OR [aOR] = 9.89 [95% CI 3.62–26.9]), followed by nasal cannula (aOR = 3.97 [95% CI 2.60–6.30]) and high-flow nasal cannula (aOR = 3.64 [95% CI 1.58–7.71]) use during primary SARS-CoV-2 infection, heart failure or hypertension (aOR = 2.09 [95% CI 1.47–2.98]), and male sex (aOR = 1.44 [95% CI 1.03–1.99]; Table 3; reference group: unhospitalized primary infection patients). In a subanalysis using only hospitalized patients (WHO score 4–7), the association of IMV with diffusion impairment and severe or moderate restriction was comparable with whole-cohort results (aOR = 9.56 [95% CI 3.22–32.6] vs. reference group of hospitalized room-air patients; Supplemental Table 5). To evaluate the effect of patients with preexisting pulmonary comorbidities, further sensitivity testing was performed (Supplemental Tables 6 and 7). Overall, the results show that IMV still conferred the greatest risk for development of pulmonary long COVID with diffusion impairment and severe or moderate restriction (Supplemental Table 6, cohort sans patients with pulmonary comorbidities vs. reference group of nonhospitalized patients aOR = 13.1 [95% CI 4.41–48.1]; Supplemental Table 7, cohort sans patients with pulmonary comorbidities vs. reference group of hospitalized room-air patients aOR = 15.2 [95% CI 4.72–68.1]).

### Assessment of CT imaging and pathology.

**Figure 3:**
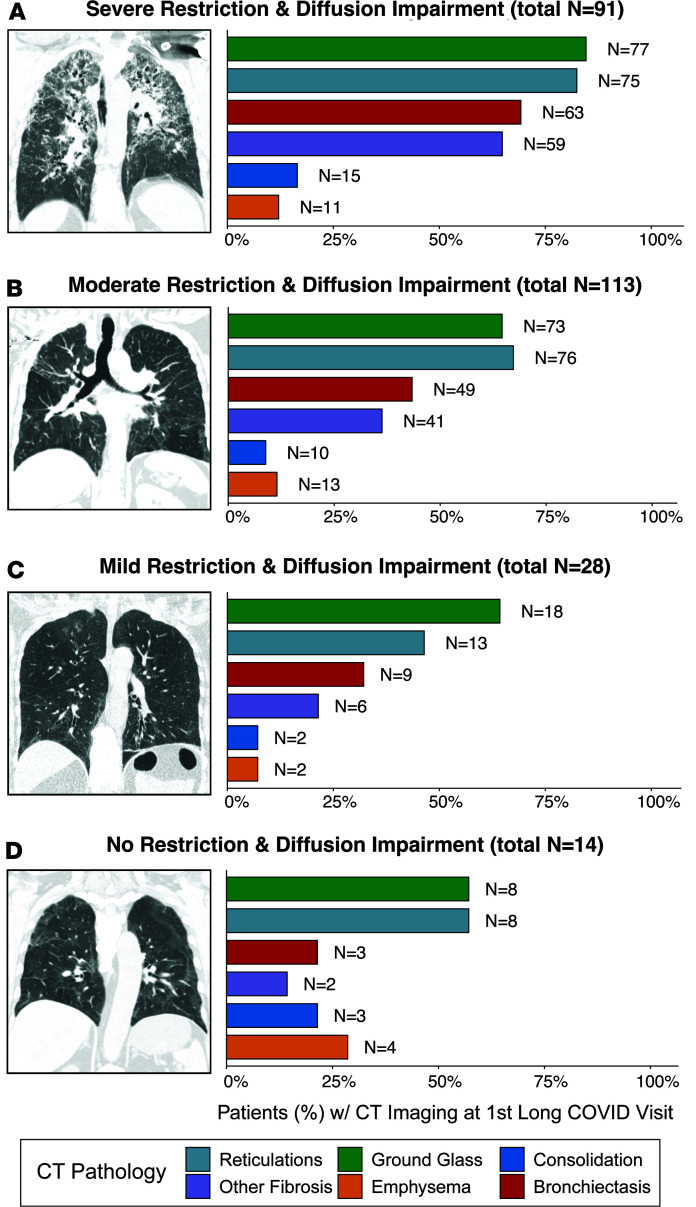
CT image findings in pulmonary long COVID with diffusion-impaired restriction. (**A**–**D**) Representative CT images of pulmonary long COVID patients with diffusion impairment (DLCO ≤ 80%) with severe (TLC ≤ 50%), moderate (TLC 51%–70%), mild (TLC 71%–80%), and no restriction (TLC > 80%) assessed at the first long COVID clinic visit. Corresponding bar charts of CT pathology (%, *n* = patients) are displayed for each group. (Note: architectural distortion, traction bronchiectasis, and honeycombing are termed “Other Fibrosis”).

CT imaging was performed on a total of 308 patients (33%; *n* = 246 diffusion-impaired subgroup, *n* = 62 diffusion-normal subgroup; Tables 1 and 2). CT pathology and increased lung involvement were predominantly found in diffusion-impaired patients with severe or moderate restriction. CT scoring of images taken within 6 months of the first long COVID visit identified ground glass opacities (85%), reticulations (82%), bronchiectasis (69%), and fibrotic changes (65%) as the defining pathologies in the majority of diffusion-impaired severe restriction patients (Figure 3A and Supplemental Table 2). A similar pathologic profile was found in diffusion-impaired patients with moderate restriction (Figure 3B). Fibrotic changes were greatly reduced in patients with mild restriction and diffusion impairment (Figure 3C) and those individuals without restriction and diffusion impairment (Figure 3D). Univariable and multivariable logistic regression modeling was performed to determine whether CT pathologies were associated with increased odds of developing pulmonary long COVID with severe or moderate restriction (Supplemental Table 8). The significant unadjusted ORs for developing severe restriction were associated with ground glass opacities (OR = 4.11 [95% CI 2.50–6.96]), reticulations (OR = 5.49 [95% CI 3.24–9.35]), fibrotic changes (OR = 5.27 [95% CI 3.10–9.60]), bronchiectasis (OR = 5.27 [95% CI 3.10–9.60]), and consolidation (OR = 2.35 [95% CI 1.02–9.06]); however, only reticulations (aOR = 2.12 [95% CI 1.01–4.34]) maintained a significance in multivariable modeling (Supplemental Table 8).

## Discussion

Our current understanding of persistent pulmonary defects from SARS-CoV-2 infection are primarily derived from prospective follow-up studies assessing patient outcomes after hospitalization with acute COVID-19. Gradual recovery of impaired diffusion capacity (DLCO ≤ 80%) and radiographic evidence of fibrotic pulmonary tissue are among the most commonly reported observations (7–10, 19–23). Although these studies offer key insights into the trajectory of acute COVID recovery, their prospective study design and inclusion of patients without post-COVID symptoms limit the applicability of these findings to long COVID patients with persistent dyspnea, cough, and chest discomfort. To address this knowledge gap, we leveraged a large, demographically diverse cohort comprised exclusively of long COVID patients with persistent pulmonary symptoms. For this cohort, we aligned robust medical record data requiring no imputation, quantitative CT imaging, and longitudinal PFT to identify the characteristics and determinants of pulmonary long COVID. Our study establishes a clear association between burden of acute COVID disease and the development of persistent lung restriction with diffusion impairment. Collectively, these results represent a previously undescribed pathology for long COVID that can be readily measured with PFTs and provides an opportunity to better study the postviral interplay of pulmonary symptoms with changes in lung physiology.

Demographic risk factors associated with the development of pulmonary long COVID with diffusion impairment and severe or moderate restriction included male sex (OR = 1.63 [95% CI 1.23–2.23]; aOR = 1.22 [95% CI 0.81–1.89]) and preexisting heart failure and hypertension (OR = 2.54 [95% CI 1.98–3.32]; aOR = 2.09 [95% CI 1.47–2.98]; Table 3). These findings differ from a recent meta-analysis of multiorgan long COVID symptoms that noted an elevated risk in females (aOR = 1.56) and individuals over 40 years of age (aOR = 1.21) (24). Our findings emphasize differences between the assessments obtained from broad symptom observations and physiological readouts in pinpointing at-risk populations. In long COVID patients complaining of prolonged pulmonary symptoms, prior studies have suggested that dyspnea is a major feature and that symptoms can persist for months after initial infection (9, 25). Our study affirmed this observation, with 78% (*n* = 725) of the UAB pulmonary long COVID cohort identifying dyspnea as the primary symptom, followed by 18% (*n* = 167) with cough and 4% (*n* = 37) with chest discomfort (Tables 1 and 2). Notably, the complaint of dyspnea remained consistent (>70%) across all degrees of diffusion impairment, restriction, and levels of lung involvement seen on CT imaging (Tables 1 and 2 and Supplemental Table 2). This suggests symptoms alone do not provide sufficient granularity to identify distinct endotypes of pulmonary long COVID, thereby highlighting the importance of incorporating routine PFTs in the diagnostic evaluation of this patient population.

Our study demonstrates that the severity of hypoxia during primary SARS-CoV-2 infection is a critical factor in the development of pulmonary long COVID with persistent diffusion impairment and restriction (Table 3). Notably, the post-hospitalization PFT impairments in pulmonary long COVID contrast with previously described post-ARDS findings in non-COVID patients, where patients predominately present with isolated diffusion impairment that improves to normal levels over a 6-month to 1-year period and limited lung restriction at any time point (26–29). In addition to marked differences in lung physiology, the presence of reticulations, bronchiectasis, ground glass opacities, and fibrotic changes in pulmonary long COVID CT images are distinct from ARDS, which has been predominantly described by the presence of ground glass opacities and reticulations (28). Cumulatively, our physiologic and radiographic evidence suggest that pulmonary long COVID is a profibrotic disease process that is distinct from ARDS; however, future studies are warranted and needed to further elucidate the differences.

The biologic mechanisms underlying these symptomatic, physiologic, and radiographic changes are poorly understood, but there is increasing evidence that profibrotic interstitial lung changes are occurring in dyspneic patients as early as 1 month after COVID infection (18, 30–32). From a molecular perspective, several independent lines of evidence have shown that altered immune function, dysregulation of systemic neutrophilic signatures, and persistent inflammation and presence of viral antigens are associated with long COVID (33–36). While few studies have been conducted in the long COVID lung, a prior spatial transcriptomic lung autopsy study from COVID-19 acute lung injury demonstrates a distinct fibroproliferative phenotype relative to influenza infection (37). If true, pulmonary fibrosis therapeutics like nintedanib and pirfenidone, which have been sparingly used in post-COVID–associated fibrosis, may be uniquely suited for the subset of pulmonary long COVID patients with diffusion-impaired restriction (38–40). This report provides evidence for a distinct endotype of pulmonary long COVID and emphasizes the need to stratify patients with PFTs for targeted therapeutic and clinical management.

This study has limitations. Due to the retrospective nature of the cohort, PFTs were not taken before or during primary SARS-CoV-2 infection, and therefore could not be compared to measurements taken at the first long COVID clinic visit. While this large, demographically diverse cohort offers a unique opportunity to characterize pulmonary presentations of long COVID, patients presented to the long COVID clinic at different time intervals after primary COVID infection, and follow-ups were limited to attended visits. Similarly, inherent bias toward more severe disease is present in patients who had CT imaging performed. Lastly, this study is limited by a lack of quality-of-life measurements such as the 6-minute walk test performed during clinic visits. Despite these limitations, the combination of an objective physiologic metric in longitudinal PFTs and a previously established long COVID symptomatic signature provide an important foundation for future long COVID studies.

Although dyspnea is present in a majority of pulmonary long COVID patients, reported symptoms were not representative of the degree of diffusion impairment and restriction across the cohort (41). The additional granularity provided with PFTs highlights the utility of a broadly available clinical test to identify pulmonary endotypes associated with persistent physiologic impairment. These insights underscore the need for medical providers to incorporate PFT measurements as a routine step for evaluating long COVID patients with pulmonary complaints. Informed stratification of patients experiencing pulmonary long COVID is critical, as this population is likely to require high utilization of health care services and would likely benefit from early therapeutic interventions (42).

## Methods

### Sex as a biological variable.

The retrospective design of this study had no exclusion criteria based on patient biological sex. After exclusion (Figure 1), our cohort consisted of 66% female (*n* = 609) and 34% male (*n* = 302) patients, reflecting previously reported biases toward development of long COVID symptoms in female patients (1–3). To evaluate biological sex as a risk factor for developing pulmonary long COVID, patient biological sex was used as a variable in univariable and multivariable outcome modeling (Table 3) and subsequent model sensitivity testing (Supplemental Tables 5–7 and Supplemental Table 10).

### Study design and population.

This single-center, retrospective cohort study was performed among adult patients (≥18 years) with a positive COVID-19 PCR and/or rapid antigen test during the study window (March 2020 to August 2023), followed by self- or physician referral to the UAB Post-COVID Pulmonary Clinic for chief complaint of unresolved respiratory symptoms categorized as dyspnea, cough, or chest discomfort (note: the presence of extrapulmonary symptoms was not an exclusion criteria; however, only a single pulmonary chief complaint was recorded). Pulmonary long COVID was defined as pulmonary symptoms persisting for 1 or more months that had developed 28 days or more after resolution of primary SARS-CoV-2 infection. PFTs were performed on all patients (if able to physically complete the exam) referred for in-person clinic visit. Chest CT imaging orders were made at the discretion of the attending physician. Baseline (first visit) PFTs and CT scans were defined by the first date of the measurement within a window of 14 days prior to and 6 months after the patient’s first long COVID clinic visit date. Follow-up measurements (termed second and third visit) were restricted to the study window and had to occur after the first-encounter measurement. The cohort was stratified by percentage predicted DLCO (normal, DLCO > 80%; impaired, DLCO ≤ 80%) and percentage predicted TLC in accord with the American Thoracic Society consensus definition for restrictive lung disease. Lung restriction subgroupings were (a) no-restriction TLC greater than 80%, (b) mild-restriction TLC 71%–80%, (c) moderate restriction TLC 51–70%, and (d) severe-restriction TLC ≤ 50% (43–44). All pulmonary function studies were conducted utilizing the same equipment (Vyntus system from Vyaire Medical Incorporated). For further information on cohort stratification by primary SARS-CoV-2 infection severity, see Supplemental Table 9.

### Patient variables extracted from electronic medical record and radiographic images.

Clinical variables were extracted for all patients in the cohort including: advanced age (≥ 65 years), biological sex, elevated BMI (≥ 30), smoking history (never, former, or current smoker), pre-COVID vaccination status, ICU admission, COVID-19 severity (assessed using the WHO score system representing maximum oxygen therapy support required), and therapeutics (dexamethasone, remdesivir, days on oxygen a specific oxygen support device) during primary infection, SARS-CoV-2 variant, and months from primary infection to first long COVID clinic visit (45). Pre-COVID comorbidities (renal, pulmonary, heart failure or hypertension, diabetes, obstructive sleep apnea, and immunosuppression) were determined by physician review of medical records. CT imaging was assessed by 2 blinded cardiothoracic radiologists for the presence or absence of 6 pulmonary imaging findings: lung consolidation, ground-glass opacities, reticulations, other fibrotic-like changes (i.e., architectural distortion, traction bronchiectasis and honeycombing; termed “other fibrosis”), bronchiectasis, and emphysema (see Supplemental Table 3 for 2-reader similarity evaluation and Supplemental Table 11 for analysis of PFT and CT imaging in a subcohort of patients with 3 consecutive follow-up visits). An overall severity score was determined using a previously defined image scoring system quantifying abnormalities in all 5 lung lobes, with scores ranging from 0 (no involvement) to 25 (multilobe involvement) (46). Detailed information on variables extracted can be found in Supplemental Table 1.

### Statistics.

Cohort statistics are reported with mean ± SD or median [Q1–Q3] in Tables 1 and 2. Alluvial diagrams were used to assess relative DLCO and TLC improvement or decline in patients with 3 consecutive follow-up visits (Figure 2C). Logistic regression models (unadjusted variable and multivariable adjusted) were used to discover risk factors for developing diffusion impairment (DLCO ≤ 80%) with severe or moderate restriction (TLC ≤ 70%) at first long COVID clinic visit (Table 3; see Supplemental Table 1 for details on model variables and Supplemental Tables 5–7 and Supplemental Table 10 for model sensitivity testing). All modeling results are reported as either unadjusted ORs or aORs with bootstrapped (*n* = 1000 iterations) 95% CIs. All statistical analyses were performed using R (version 4.2, R Foundation; https://www.R-project.org/).

### Study approval.

The study was approved by the IRB of the UAB (IRB nos. 300006291 and 300006205).

### Data availability.

Select deidentified data from the UAB 2020–2022 pulmonary long COVID cohort can be made available upon request with IRB approval and signing of institutional data use agreements.

## Author contributions

MJP, NE, DB, SG, EBL, AG, SWR, DR, KP, MLL, PM, and MM conceived the project, prepared the main text, and oversaw the UAB cohort creation. MJP processed and analyzed electronic medical record data to create all tables and figures in the main text and supplement of this manuscript. DB analyzed and scored CT images. SG analyzed and scored a subset of CT images. NE oversaw IRB approval from the UAB. NE, MM, DR, and AG obtained funding and supervised the overall study. All coauthors reviewed and approved the final version of the manuscript.

## Supplementary Material

Supplemental data

ICMJE disclosure forms

Supporting data values

## Figures and Tables

**Table 3:**
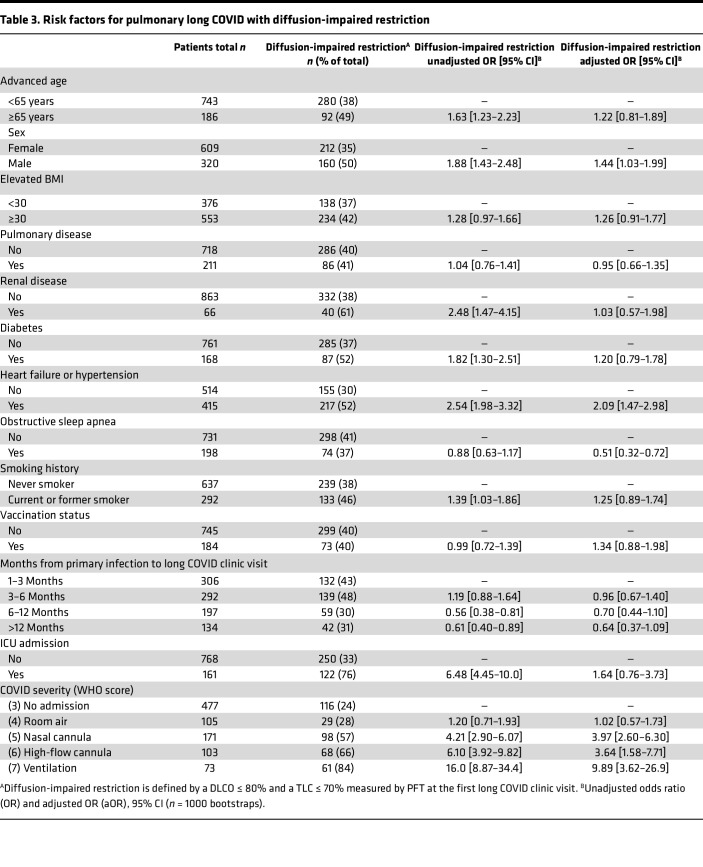
Risk factors for pulmonary long COVID with diffusion-impaired restriction

**Table 2:**
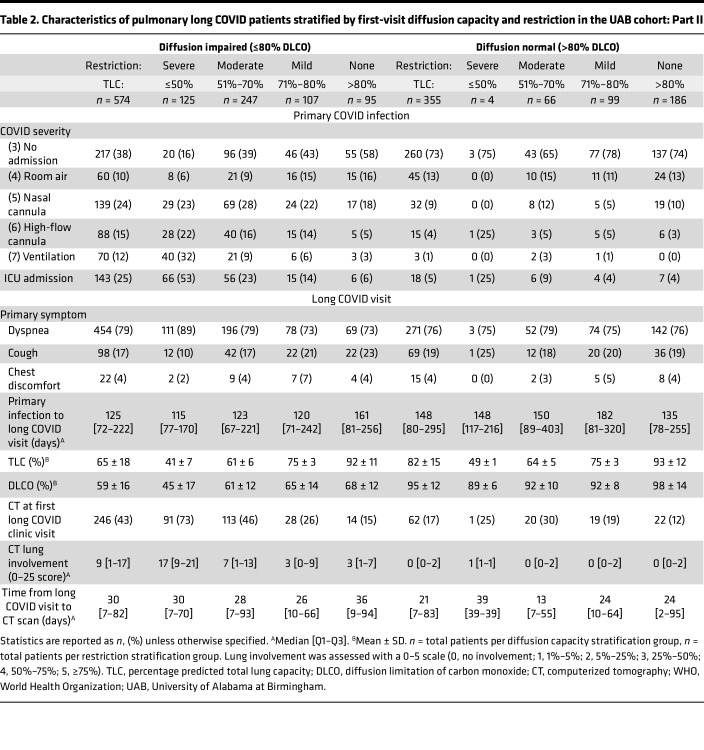
Characteristics of pulmonary long COVID patients stratified by first-visit diffusion capacity and restriction in the UAB cohort: Part II

**Table 1:**
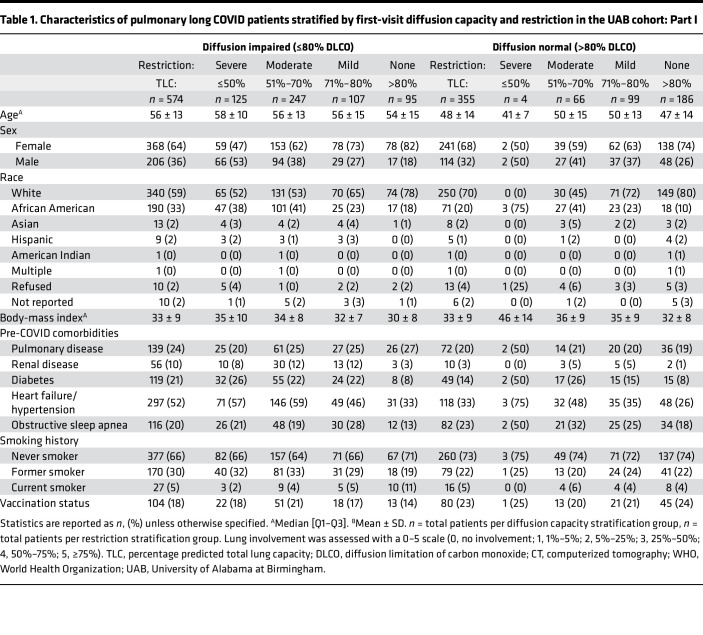
Characteristics of pulmonary long COVID patients stratified by first-visit diffusion capacity and restriction in the UAB cohort: Part I

## Version 1 04/23/2024

In-Press Preview

## Version 2 05/22/2024

Electronic publication
