# Supplementary material for: Characteristics and determinants of pulmonary long COVID
Source: JCI Insight. 2024 Apr 23;9(10):e177518. doi: 10.1172/jci.insight.177518 (PMC11141907; doi:10.1172/jci.insight.177518)
Supplement: Supplemental data [file jciinsight-9-177518-s248.pdf]

SUPPLEMENTAL MATERIALS

Appendix

Supplemental Table 1: Patient Characteristics Extracted from Electronic Medical Record (EMR) Data and CT Image Analysis used for Outcome Modeling 2
Supplemental Table 2: Supplemental Cohort Characteristics of Pulmonary Long COVID Patients Stratified by Diffusion Capacity and Restriction in the UAB Cohort 3
Supplemental Table 3: CT Analysis Reader Agreement of Images taken within 6-months of the 1st Pulmonary Long COVID Clinic Visit 5
Supplemental Table 4: Longitudinal Pulmonary Function Testing Summary Statistics for 1st, 2nd, and 3rd, Pulmonary Long COVID Clinic Visits Stratified by 1st Visit Diffusion Impairment and Restriction 6
Supplemental Table 5: Outcome Modeling of Pulmonary Long COVID with Diffusion Impaired Restriction in Hospitalized COVID Patients (Sensitivity Testing) 8
Supplemental Table 6: Outcome Modeling of Pulmonary Long COVID with Diffusion Impaired Restriction in Patients without Pre-Existing Pulmonary Comorbidities 10
Supplemental Table 7: Outcome Modeling of Pulmonary Long COVID with Diffusion Impaired Restriction in Hospitalized COVID Patients without Pre-Existing Pulmonary Comorbidities (Sensitivity Testing) 12
Supplemental Table 8: Outcome Modeling of Pulmonary Long COVID with Diffusion Impaired Restriction from CT Image Pathology 14
Supplemental Table 9: Characteristics of Pulmonary Long COVID Patients Stratified by Primary SARS-CoV-2 Infection Severity in the UAB Cohort 15
Supplemental Table 10: Outcome Modeling of Pulmonary Long COVID with Diffusion Impaired Restriction (Sensitivity Testing with NICE Definition of Long COVID) 19
Supplemental Table 11: Pulmonary Function Test and CT Image Comparison for Pulmonary Long COVID Patients with 3-Followup Visits (n=17 patients) 21

Supplemental Table 1: Patient Characteristics Extracted from Electronic Medical Record (EMR) Data and CT Image Analysis used for Outcome Modeling

Variable Type	Time Point	Variable Name	Variable Description
Electronic Medical Record & Chart Review	Before Primary SARS-CoV-2 Infection	Diabetes (Type I or II)	Categorical (yes or no)
		Renal Disease	Categorical (yes or no)
		Pulmonary Disease	Categorical (yes or no)
		Hypertension or Heart Failure	Categorical (yes or no)
		Obstructive Sleep Apnea	Categorical (yes or no)
		Vaccination Status (1st dose)	Categorical (yes or no)
		Smoking History	Categorical (current, former, or never)
	During Primary SARS-CoV-2 Infection	SARS-CoV-2 Infection Wave[a]	Categorical (Alpha, Delta, Omicron)
		WHO Severity Score (Worst score during entire hospitalization)	Categorical (3-7)<br>- (3) No Hospital Admission<br>- (4) Admitted, Room Air<br>- (5) Admitted, Nasal Cannula<br>- (6) Admitted, High-Flow Nasal Cannula (>5L/min)<br>- (7) Admitted, Invasive Mechanical Ventilation
	1st Pulmonary Long COVID Clinic Visit	Age	Continuous (years)
		Body-Mass Index	Continuous (kg/m^2)
		Biological Sex	Categorical (male or female)
		Time from primary infection to 1st Long COVID clinic visit	Continuous (months)
CT Image Analysis	Image taken within 6-months of 1st Pulmonary Long COVID Clinic Visit	Right Upper Lung Involvement[b]	Continuous (0-5)
		Right Middle Lung Involvement[b]	Continuous (0-5)
		Right Lower Lung Involvement[b]	Continuous (0-5)
		Left Upper Lung Involvement[b]	Continuous (0-5)
		Left Lower Lung Involvement[b]	Continuous (0-5)
		Cumulative Lung Involvement[b]	Continuous (0-25)
		Lung Consolidation	Categorical (yes or no)
		Ground-Glass Opacities	Categorical (yes or no)
		Reticulations	Categorical (yes or no)
		Bronchiectasis	Categorical (yes or no)
		Emphysema	Categorical (yes or no)
		Other Fibrosis (Architectural distortions, traction bronchiectasis, honeycombing)	Categorical (yes or no)

[a]SARS-CoV-2 waves defined by following date ranges (Alpha: 01/01/2020-05/31/2021, Delta:06/01/2021-01/01/2022, Omicron: 01/02/2022-08/01/2023)
[b]Lung Involvement was assessed with a 0-5 scale (0=no involvement, 1=1-5%, 2=5-25%, 3=25-50%, 4=50-75%, 5≥75%)

Supplemental Table 2: Supplemental Cohort Characteristics of Pulmonary Long COVID Patients Stratified by Diffusion Capacity and Restriction in the UAB Cohort

	Diffusion Impaired (≤80% DLCO)					Diffusion Normal (>80% DLCO)				
	Restriction:	Severe	Moderate	Mild	None	Restriction:	Severe	Moderate	Mild	None
	TLC:	≤50%	51-70%	71-80%	>80%	TLC:	≤50%	51-70%	71-80%	80%
	N=574	n=125	n=247	n=107	n=95	N=355	n=4	n=66	n=99	n=186
Primary COVID Infection										
SARS-CoV-2 Variant Wave										
Alpha Wave	357 (62)	67 (54)	154 (62)	69 (64)	67 (71)	225 (63)	1 (25)	48 (73)	59 (60)	117 (63)
Delta Wave	130 (23)	32 (26)	57 (23)	25 (23)	16 (17)	74 (21)	2 (50)	10 (15)	22 (22)	40 (22)
Omicron Wave	87 (15)	26 (21)	36 (15)	13 (12)	12 (13)	56 (16)	1 (25)	8 (12)	18 (18)	29 (16)
Immunosuppressed	137 (38)	38 (36)	68 (45)	18 (30)	13 (33)	18 (19)	0 (0)	5 (22)	4 (18)	9 (18)
Unknown	*218*	*20*	*97*	*46*	*55*	*260*	*3*	*43*	*77*	*137*
Remdesivir	178 (46)	60 (55)	78 (50)	27 (42)	13 (24)	29 (19)	1 (100)	6 (17)	6 (15)	16 (21)
Unknown	*188*	*15*	*90*	*42*	*41*	*200*	*3*	*31*	*58*	*108*
Dexamethasone	239 (62)	79 (72)	104 (65)	37 (57)	19 (35)	45 (29)	1 (100)	14 (40)	10 (24)	20 (26)
Unknown	*186*	*15*	*88*	*42*	*41*	*200*	*3*	*31*	*58*	*108*
COVID Severity (WHO Score)										
(3) No Admission	217 (38)	20 (16)	96 (39)	46 (43)	55 (58)	260 (73)	3 (75)	43 (65)	77 (78)	137 (74)
(4) Room Air	60 (10)	8 (6)	21 (9)	16 (15)	15 (16)	45 (13)	0 (0)	10 (15)	11 (11)	24 (13)
(5) Nasal Cannula	139 (24)	29 (23)	69 (28)	24 (22)	17 (18)	32 (9)	0 (0)	8 (12)	5 (5)	19 (10)
(6) High-Flow Cannula	88 (15)	28 (22)	40 (16)	15 (14)	5 (5)	15 (4)	1 (25)	3 (5)	5 (5)	6 (3)
(7) Ventilation	70 (12)	40 (32)	21 (9)	6 (6)	3 (3)	3 (1)	0 (0)	2 (3)	1 (1)	0 (0)
Nasal Cannula Time (days)[1]	7 [4-11]	8 [6-18]	7 [4-10]	4 [4-7]	7 [3-11]	5 [3-6]	6 [6-6]	5 [3-6]	5 [4-7]	5 [4-6]
Unknown	*365*	*65*	*147*	*78*	*75*	*318*	*3*	*57*	*92*	*166*
High-flow Nasal Cannula Time (days)[1]	5 [3-8]	8 [3-9]	4 [2-6]	5 [2-9]	4 [2-5]	5 [2-9]	9 [9-9]	2 [2-2]	11 [8-13]	5 [3-9]
Unknown	*493*	*96*	*213*	*95*	*89*	*345*	*3*	*64*	*97*	*181*
Ventilation Time (days)[1]	18 [8-35]	27 [9-41]	13 [5-19]	9 [7-11]	24 [16-28]	14 [10-19]	-	14 [10-19]	-	-
Unknown	*516*	*90*	*231*	*103*	*92*	*353*	*4*	*64*	*99*	*186*

Supplemental Table 2: Supplemental Cohort Characteristics of Pulmonary Long COVID Patients Stratified by Diffusion Capacity and Restriction in the UAB Cohort

	Diffusion Impaired (≤80% DLCO)					Diffusion Normal (>80% DLCO)				
	Restriction:	Severe	Moderate	Mild	None	Restriction:	Severe	Moderate	Mild	None
	TLC:	≤50%	51-70%	71-80%	>80%	TLC:	≤50%	51-70%	71-80%	80%
	N=574	n=125	n=247	n=107	n=95	N=355	n=4	n=66	n=99	n=186
CT Scoring Evaluations[2]										
Cumulative Involvement (0-25)[1]	9 [1-17]	17 [9-21]	7 [1-13]	3 [0-9]	3 [1-7]	0 [0-2]	1 [1-1]	0 [0-2]	0 [0-2]	0 [0-2]
Right Upper Lung (0-5)	2 [0-3]	3 [1-4]	1 [0-3]	0 [0-2]	0 [0-1]	0 [0-0]	0 [0-0]	0 [0-0]	0 [0-0]	0 [0-0]
Right Middle Lung (0-5)	2 [0-4]	3 [2-5]	1 [0-3]	0 [0-2]	0 [0-1]	0 [0-0]	0 [0-0]	0 [0-0]	0 [0-0]	0 [0-0]
Right Lower Lung (0-5)	2 [0-4]	3 [1-4]	1 [0-3]	1 [0-2]	1 [1-2]	0 [0-1]	0 [0-0]	0 [0-1]	0 [0-1]	0 [0-1]
Left Upper Lung (0-5)	2 [0-3]	3 [2-4]	1 [0-2]	0 [0-2]	0 [0-1]	0 [0-0]	0 [0-0]	0 [0-0]	0 [0-1]	0 [0-0]
Left Lower Lung (0-5)	2 [1-4]	3 [2-4]	1 [0-3]	1 [0-2]	1 [0-2]	0 [0-1]	1 [1-1]	0 [0-1]	0 [0-0]	0 [0-1]
*Unknown**	*328*	*34*	*134*	*79*	*81*	*293*	*3*	*46*	*80*	*164*
CT Pathology										
Ground Glass Opacities	176 (72)	77 (85)	73 (65)	18 (64)	8 (57)	16 (26)	1 (100)	4 (20)	5 (26)	6 (27)
Reticulations	172 (70)	75 (82)	76 (67)	13 (46)	8 (57)	15 (24)	0 (0)	6 (30)	5 (26)	4 (18)
Other Fibrosis	108 (44)	59 (65)	41 (36)	6 (21)	2 (14)	5 (8)	0 (0)	2 (10)	1 (5)	2 (9)
Bronchiectasis	124 (50)	63 (69)	49 (43)	9 (32)	3 (21)	8 (13)	0 (0)	3 (15)	2 (11)	3 (14)
Consolidation	30 (12)	15 (16)	10 (9)	2 (7)	3 (21)	1 (2)	0 (0)	0 (0)	0 (0)	1 (5)
Emphysema	30 (12)	11 (12)	13 (12)	2 (7)	4 (29)	4 (6)	0 (0)	1 (5)	2 (11)	1 (5)
*Unknown**	*328*	*34*	*134*	*79*	*81*	*293*	*3*	*46*	*80*	*164*
Expiratory Scan CT Pathology										
Gas Trapping (Expiratory Only)[3]	118 (67)	40 (57)	59 (74)	16 (76)	3 (60)	24 (69)	1 (100)	8 (53)	10 (83)	5 (71)
Unknown	*398*	*55*	*167*	*86*	*90*	*320*	*3*	*51*	*87*	*179*

[1]Statistics are reported as n, (%) unless otherwise specified as Median [Q1-Q3]; N=total patients per diffusion capacity stratification group, n=total patients per restriction stratification group
[2]Lung Involvement was assessed with a 0-5 scale (0=no involvement, 1=1-5%, 2=5-25%, 3=25-50%, 4=50-75%, 5≥75%)
[3]Gas trapping was evaluated in a small subset of patients with expiratory CT scans and was therefore not used as an outcome modeling variable.
*Unknown counts denoted with an asterix represent the count for all variables in the category (i.e. CT Scoring Evaluations, Median [IQR] and CT Pathology)
Abbreviations: TLC, percent predicted total lung capacity; DLCO, diffusion limitation of carbon monoxide; CT, computerized tomography; WHO, World Health Organization; COVID, coronavirus disease; UAB, University of Alabama at Birmingham

Supplemental Table 3: CT Analysis Reader Agreement of Images taken within 6-months of the 1st Pulmonary Long COVID Clinic Visit

	Reader #1, N=91	Reader #2, N = 91	p-value
CT Score - Right Upper Lung (0-5)[3]			0.68[1]
0	41 (45)	36 (40)	
1	14 (15)	21 (23)	
2	11 (12)	11 (12)	
3	9 (10)	7 (8)	
4	10 (11)	7 (8)	
5	6 (7)	9 (10)	
CT Score - Right Middle Lung (0-5)[3]			0.38[1]
0	43 (47)	36 (40)	
1	10 (11)	20 (22)	
2	11 (12)	10 (11)	
3	9 (10)	7 (8)	
4	12 (13)	9 (10)	
5	6 (7)	9 (10)	
CT Score - Right Lower Lung (0-5)[3]			0.96[1]
0	32 (35)	32 (35)	
1	20 (22)	21 (23)	
2	9 (10)	9 (10)	
3	13 (14)	9 (10)	
4	11 (12)	12 (13)	
5	6 (7)	8 (9)	
CT Score - Left Upper Lung (0-5)[3]			0.043[1]
0	43 (47)	37 (41)	
1	7 (8)	20 (22)	
2	13 (14)	11 (12)	
3	12 (13)	7 (8)	
4	12 (13)	7 (8)	
5	4 (4)	9 (10)	
CT Score - Left Lower Lung (0-5)[3]			0.35[1]
0	34 (37)	30 (33)	
1	17 (19)	21 (23)	
2	14 (15)	7 (8)	
3	10 (11)	14 (15)	
4	11 (12)	9 (10)	
5	5 (5)	10 (11)	
CT Score - Cumulative Involvement[3] (0-25), Median [Q1-Q3]	5 [0-15]	6 [1-15]	0.60[2]
Pathologies Present			
Ground Glass Opacities	57 (63)	66 (73)	0.15[1]
Reticulations	54 (59)	37 (41)	0.012[1]
Other Fibrosis	28 (31)	44 (48)	0.015[1]
Bronchiectasis	36 (40)	40 (44)	0.55[1]
Consolidation	9 (10)	12 (13)	0.49[1]
Emphysema	12 (13)	12 (13)	>0.99[1]

[1]Pearson's Chi-squared test
[2]Wilcoxon rank sum test
[3]Lung Involvement was assessed with a 0-5 scale (0=no involvement, 1=1-5%, 2=5-25%, 3=25-50%, 4=50-75%, 5≥75%)
Statistics are reported as n (% of total) unless otherwise stated.

Supplemental Table 4: Longitudinal Pulmonary Function Testing Summary Statistics for 1st, 2nd, and 3rd, Pulmonary Long COVID Clinic Visits Stratified by 1st Visit Diffusion Impairment and Restriction

	Diffusion Impaired (≤80% DLCO)					Diffusion Normal (>80% DLCO)				
Percent Predicted PFT (%)[a]	Restriction: TLC:	Severe ≤50%	Moderate 51-70%	Mild 71-80%	None >80%	Restriction: TLC:	Severe ≤50%	Moderate 51-70%	Mild 71-80%	None >80%
1st Long COVID Clinic Visit[b]	N=574	n=125	n=247	n=107	n=95	N=335	n=4	n=66	n=99	n=186
Total Lung Capacity (TLC)	64[24] 65±18	43[10] 41±7	62[9] 61±6	75[5] 75±3	87[15] 92±11	81[18] 82±15	49[0] 49±1	65[8] 64±5	75[5] 75±3	90[12] 93±12
Diffusion Capacity (DLCO)	63[22] 59±16	44[27] 45±17	63[17] 61±12	67[16] 65±14	71[13] 68±12	93[16] 95±12	89[6] 89±6	89[10] 92±10	92[12] 92±8	96[19] 98±14
Forced Vital Capacity (FVC)	75[25] 74±18	52[15] 51±12	74[17] 74±12	83[16] 84±12	91[18] 91±15	92[19] 93±14	79[8] 76±9	82[11] 81±10	88[13] 88±10	99[17] 100±13
Residual Volume (RV)	42[31] 47±31	26[18] 25±13	39[23] 40±17	54[28] 56±21	74[47] 84±45	51[34] 55±31	1[3] 3±3	36[22] 37±19	49[25] 47±19	62[35] 66±34
Unknown	*6*	*5*	*0*	*1*	*0*	*16*	*1*	*8*	*7*	*0*
Forced Expiratory Volume 1-second (FEV1)	75[25] 74±19	53[17] 55±13	74[16] 74±15	85[17] 84±15	88[18] 87±17	91[18] 91±15	77[12] 69±20	84[12] 83±10	87[16] 88±13	96[17] 97±15
Unknown	-	-	-	-	-	*1*	*0*	*0*	*0*	*1*
Forced Mid-Expiratory Flow (FEF 25/75)	83[47] 87±40	83[53] 86±45	82[47] 86±38	90[48] 92±37	83[52] 88±39	90[39] 93±32	71[26] 64±28	91[37] 94±27	88[44] 92±34	91[37] 94±32
Unknown	*1*	*0*	*0*	*0*	*1*	*2*	*0*	*1*	*1*	*0*
2nd Long COVID Clinic Visit[b]	N=282	n=77	n=132	n=45	n=28	N=96	n=3	n=27	n=29	n=37
Total Lung Capacity (TLC)	65[18] 65±15	51[14] 51±14	65[15] 66±11	70[15] 73±10	81[25] 82±18	78[20] 79±13	64[3] 64±4	70[13] 70±10	78[14] 79±10	88[15] 87±12
Unknown	*24*	*8*	*7*	*4*	*5*	*13*	*1*	*1*	*6*	*5*
Diffusion Capacity (DLCO)	67[28] 66±19	50[20] 51±16	69[21] 70±16	77[22] 74±19	82[16] 76±20	89[16] 93±16	100[11] 100±15	87[12] 88±11	89[9] 90±12	95[21] 98±20
Unknown	*11*	*2*	*3*	*2*	*4*	*11*	*1*	*2*	*4*	*4*
Forced Vital Capacity (FVC)	77[23] 76±17	59[16] 61±16	80[19] 79±15	87[18] 85±12	88[26] 89±16	90[15] 90±14	76[22] 74±22	86[10] 85±9	90[9] 90±11	96[19] 94±17

Supplemental Table 4: Longitudinal Pulmonary Function Testing Summary Statistics for 1st, 2nd, and 3rd, Pulmonary Long COVID Clinic Visits Stratified by 1st Visit Diffusion Impairment and Restriction

	Diffusion Impaired (≤80% DLCO)					Diffusion Normal (>80% DLCO)				
Percent Predicted PFT (%)[a]	Restriction:<br>TLC:	Severe<br>≤50%	Moderate<br>51-70%	Mild<br>71-80%	None<br>>80%	Restriction:<br>TLC:	Severe<br>≤50%	Moderate<br>51-70%	Mild<br>71-80%	None<br>>80%
Residual Volume (RV)	41[28]<br>43±24	30[26]<br>33±22	43[26]<br>44±22	44[29]<br>49±21	49[43]<br>56±33	55[33]<br>52±28	33[30]<br>33±42	45[27]<br>49±27	47[31]<br>48±23	61[36]<br>57±30
Unknown	*29*	*8*	*12*	*4*	*5*	*16*	*1*	*3*	*7*	*5*
Forced Expiratory Volume 1-second (FEV1)	77[28]<br>76±19	61[18]<br>65±18	80[26]<br>78±18	87[19]<br>85±14	86[19]<br>86±20	88[17]<br>88±16	72[30]<br>65±30	86[13]<br>86±10	86[15]<br>88±11	91[20]<br>91±19
Unknown	-	-	-	-	-	*1*	*0*	*0*	*0*	*1*
Forced Mid-Expiratory Flow (FEF 25/75)	90[53]<br>91±42	93[63]<br>97±46	86[50]<br>88±43	96[41]<br>94±32	89[53]<br>90±42	85[38]<br>89±31	62[38]<br>54±39	95[42]<br>101±30	86[38]<br>87±22	77[35]<br>84±34
3rd Long COVID Clinic Visit[b]	N=114	n=39	n=49	n=15	n=11	N=26	n=1	n=10	n=2	n=13
Total Lung Capacity (TLC)	62[21]<br>64±15	49[16]<br>55±14	65[12]<br>65±12	73[18]<br>69±12	77[21]<br>81±15	71[17]<br>72±15	56[0]<br>56±NA	71[12]<br>69±8	62[2]<br>62±2	85[28]<br>81±18
Unknown	*26*	*11*	*10*	*4*	*1*	*4*	*0*	*0*	*0*	*4*
Diffusion Capacity (DLCO)	66[25]<br>65±19	50[22]<br>54±14	69[20]<br>68±18	74[13]<br>71±13	84[28]<br>84±18	94[18]<br>90±15	93[0]<br>93±NA	94[13]<br>91±13	91[6]<br>91±8	95[27]<br>88±20
Unknown	*7*	*2*	*2*	*3*	*0*	*4*	*0*	*0*	*0*	*4*
Forced Vital Capacity (FVC)	75[23]<br>75±17	61[18]<br>63±14	79[16]<br>80±16	84[16]<br>83±11	86[17]<br>84±18	90[17]<br>89±16	79[0]<br>79±NA	91[9]<br>92±11	61[7]<br>61±10	94[19]<br>92±16
Residual Volume (RV)	37[27]<br>43±26	33[21]<br>36±22	37[26]<br>42±25	41[47]<br>41±27	58[33]<br>64±36	41[27]<br>42±20	27[0]<br>27±NA	33[19]<br>33±17	46[28]<br>46±39	53[25]<br>54±15
Unknown	*28*	*11*	*11*	*5*	*1*	*5*	*0*	*0*	*0*	*5*
Forced Expiratory Volume 1-second (FEV1)	78[26]<br>77±20	65[19]<br>67±17	80[20]<br>82±20	87[17]<br>85±11	82[36]<br>80±24	91[19]<br>88±18	79[0]<br>79±NA	93[16]<br>92±15	61[15]<br>61±21	91[13]<br>90±18
Forced Mid-Expiratory Flow (FEF 25/75)	98[61]<br>100±52	112[68]<br>103±52	95[62]<br>100±58	96[29]<br>101±29	91[63]<br>86±49	90[46]<br>93±37	79[0]<br>79±NA	95[32]<br>98±35	81[54]<br>81±76	90[47]<br>91±37

[a]Percent Predicted Pulmonary Function Testing (PFT) values are represented as median [IQR], mean ± standard deviation.
[b]N represents the overall patient count; n represents the stratified group patient count

Supplemental Table 5: Outcome Modeling of Pulmonary Long COVID with Diffusion Impaired Restriction in Hospitalized COVID Patients (Sensitivity Testing)

	Patients Total N	Diffusion Impaired Restriction[4] N (% of Total)	Diffusion Impaired Restriction Unadjusted OR [95% CI][1,2]	Diffusion Impaired Restriction Adjusted OR [95% CI][2,3]
Advanced Age				
<65 years	342	188 (55)	—	—
≥65 years	110	68 (62)	1.33 [0.85-2.06]	1.05 [0.60-1.84]
Sex				
Female	278	138 (50)	—	—
Male	174	118 (68)	2.08 [1.46-3.22]	1.82 [1.14-2.87]
Elevated BMI				
<30	160	90 (56)	—	—
≥30	292	166 (57)	1.02 [0.69-1.54]	1.17 [0.71-1.91]
Pulmonary Disease				
No	338	191 (57)	—	—
Yes	114	65 (57)	1.01 [0.65-1.57]	1.14 [0.69-1.99]
Renal Disease				
No	398	220 (55)	—	—
Yes	54	36 (67)	1.64 [0.92-3.12]	1.01 [0.51-2.45]
Diabetes				
No	344	189 (55)	—	—
Yes	108	67 (62)	1.33 [0.86-2.13]	1.21 [0.68-2.23]
Heart Failure or Hypertension				
No	203	96 (47)	—	—
Yes	249	160 (64)	2.00 [1.40-2.91]	2.12 [1.28-3.74]
Obstructive Sleep Apnea				
No	338	197 (58)	—	—
Yes	114	59 (52)	0.76 [0.52-1.21]	0.56 [0.33-0.95]
Smoking History				
Never smoker	303	166 (55)	—	—
Current or Former Smoker	149	90 (60)	1.25 [0.85-1.92]	1.04 [0.62-1.74]
Vaccination Status				
No	386	217 (56)	—	—
Yes	66	39 (59)	1.13 [0.67-1.99]	1.65 [0.77-3.39]
Months from Primary Infection to Long COVID Clinic Visit				

Supplemental Table 5: Outcome Modeling of Pulmonary Long COVID with Diffusion Impaired Restriction in Hospitalized COVID Patients (Sensitivity Testing)

	Patients Total N	Diffusion Impaired Restriction[4] N (% of Total)	Diffusion Impaired Restriction Unadjusted OR [95% CI][1,2]	Diffusion Impaired Restriction Adjusted OR [95% CI][2,3]
1-3 Months	156	91 (58)	—	—
3-6 Months	165	109 (66)	1.38 [0.91-2.21]	1.20 [0.70-2.08]
6-12 Months	73	30 (41)	0.51 [0.27-0.89]	0.56 [0.28-1.11]
>12 Months	58	26 (45)	0.58 [0.32-1.12]	0.53 [0.25-1.14]
ICU Admission				
No	291	134 (46)	—	—
Yes	161	122 (76)	3.64 [2.44-5.77]	1.56 [0.72-3.54]
COVID Severity (WHO Score)				
(4) Room Air	105	29 (28)	—	—
(5) Nasal Cannula	171	98 (57)	3.53 [2.11-6.30]	3.84 [2.14-7.62]
(6) High-Flow Cannula	103	68 (66)	5.21 [2.78-9.76]	3.76 [1.59-10.8]
(7) Ventilation	73	61 (84)	13.8 [6.78-35.1]	9.56 [3.22-32.6]

[1]Unadjusted Odds Ratio 95% confidence interval (n=1000 bootstraps)
[2]OR = Odds Ratio, CI = Confidence Interval
[3]Adjusted Odds Ratio 95% confidence interval (n=1000 bootstraps)
[4]Diffusion impaired restriction is defined by a DLCO ≤80% and a TLC ≤70% measured by PFT at the 1st Long COVID clinic visit

Supplemental Table 6: Outcome Modeling of Pulmonary Long COVID with Diffusion Impaired Restriction in Patients without Pre-Existing Pulmonary Comorbidities (Sensitivity Testing)

	Patients Total N	Diffusion Impaired Restriction[4] N (% of Total)	Diffusion Impaired Restriction Unadjusted OR [95% CI][1,2]	Diffusion Impaired Restriction Adjusted OR [95% CI][2,3]
Advanced Age				
<65 years	581	218 (38)	—	—
≥65 years	137	68 (50)	1.65 [1.10-2.48]	1.27 [0.79-2.04]
Sex				
Female	463	161 (35)	—	—
Male	255	125 (49)	1.79 [1.34-2.45]	1.39 [0.96-2.03]
Elevated BMI				
<30	292	107 (37)	—	—
≥30	426	179 (42)	1.26 [0.95-1.72]	1.37 [0.95-1.98]
Renal Disease				
No	663	251 (38)	—	—
Yes	55	35 (64)	2.87 [1.69-5.25]	1.26 [0.61-2.69]
Diabetes				
No	582	216 (37)	—	—
Yes	136	70 (51)	1.82 [1.23-2.72]	1.14 [0.71-1.89]
Heart Failure or Hypertension				
No	402	122 (30)	—	—
Yes	316	164 (52)	2.47 [1.82-3.47]	2.09 [1.39-3.17]
Obstructive Sleep Apnea				
No	567	233 (41)	—	—
Yes	151	53 (35)	0.76 [0.51-1.10]	0.42 [0.25-0.67]
Smoking History				
Never smoker	507	189 (37)	—	—
Current or Former Smoker	211	97 (46)	1.43 [1.00-2.00]	1.38 [0.92-2.00]
Vaccination Status				
No	585	237 (41)	—	—
Yes	133	49 (37)	0.86 [0.57-1.24]	1.11 [0.68-1.75]
Months from Primary Infection to Long COVID Clinic Visit				
1-3 Months	232	101 (44)	—	—
3-6 Months	227	106 (47)	1.13 [0.78-1.66]	0.89 [0.57-1.37]
6-12 Months	147	43 (29)	0.53 [0.33-0.83]	0.66 [0.39-1.12]

Supplemental Table 6: Outcome Modeling of Pulmonary Long COVID with Diffusion Impaired Restriction in Patients without Pre-Existing Pulmonary Comorbidities (Sensitivity Testing)

	Patients Total N	Diffusion Impaired Restriction[4] N (% of Total)	Diffusion Impaired Restriction Unadjusted OR [95% CI][1,2]	Diffusion Impaired Restriction Adjusted OR [95% CI][2,3]
>12 Months	112	36 (32)	0.61 [0.38-1.01]	0.66 [0.37-1.18]
ICU Admission				
No	597	196 (33)	—	—
Yes	121	90 (74)	6.02 [3.86-9.57]	1.25 [0.54-2.88]
COVID-19 Severity				
(3) No Admission	380	95 (25)	—	—
(4) Room Air	72	20 (28)	1.17 [0.63-2.02]	0.96 [0.53-1.79]
(5) Nasal Cannula	130	72 (55)	3.78 [2.50-5.70]	3.44 [2.21-5.73]
(6) High-Flow Cannula	83	54 (65)	5.67 [3.33-9.58]	3.82 [1.68-9.33]
(7) Ventilation	53	45 (85)	17.2 [8.95-45.6]	13.1 [4.41-48.1]

[1]Unadjusted Odds Ratio 95% confidence interval (n=1000 bootstraps)
[2]OR = Odds Ratio, CI = Confidence Interval
[3]Adjusted Odds Ratio 95% confidence interval (n=1000 bootstraps)
[4]Diffusion impaired restriction is defined by a DLCO ≤80% and a TLC ≤70% measured by PFT at the 1st Long COVID clinic visit

Supplemental Table 7: Outcome Modeling of Pulmonary Long COVID with Diffusion Impaired Restriction in Hospitalized COVID Patients without Pre-Existing Pulmonary Comorbidities (Sensitivity Testing)

	Patients Total N	Diffusion Impaired Restriction[4] N (% of Total)	Diffusion Impaired Restriction Unadjusted OR [95% CI][1,2]	Diffusion Impaired Restriction Adjusted OR [95% CI][2,3]
Advanced Age				
<65 years	259	141 (54)	—	—
≥65 years	79	50 (63)	1.44 [0.84-2.46]	1.06 [0.53-2.11]
Sex				
Female	205	102 (50)	—	—
Male	133	89 (67)	2.06 [1.31-3.24]	1.67 [0.96-3.01]
Elevated BMI				
<30	126	70 (56)	—	—
≥30	212	121 (57)	1.06 [0.65-1.66]	1.43 [0.78-2.71]
Renal Disease				
No	292	159 (54)	—	—
Yes	46	32 (70)	1.96 [1.00-4.20]	1.21 [0.44-3.06]
Diabetes				
No	249	135 (54)	—	—
Yes	89	56 (63)	1.45 [0.88-2.42]	1.31 [0.65-2.77]
Heart Failure or Hypertension				
No	152	71 (47)	—	—
Yes	186	120 (65)	2.11 [1.33-3.25]	2.31 [1.21-4.80]
Obstructive Sleep Apnea				
No	255	151 (59)	—	—
Yes	83	40 (48)	0.62 [0.36-1.05]	0.39 [0.21-0.76]
Smoking History				
Never smoker	235	129 (55)	—	—
Current or Former Smoker	103	62 (60)	1.25 [0.79-2.02]	1.10 [0.57-2.10]
Vaccination Status				
No	292	165 (57)	—	—
Yes	46	26 (57)	1.02 [0.53-1.97]	1.58 [0.65-3.73]
Months from Primary Infection to Long COVID Clinic Visit				
1-3 Months	114	67 (59)	—	—
3-6 Months	124	80 (65)	1.27 [0.72-2.23]	1.07 [0.54-2.02]
6-12 Months	50	22 (44)	0.55 [0.26-1.06]	0.61 [0.25-1.47]

Supplemental Table 7: Outcome Modeling of Pulmonary Long COVID with Diffusion Impaired Restriction in Hospitalized COVID Patients without Pre-Existing Pulmonary Comorbidities (Sensitivity Testing)

	Patients Total N	Diffusion Impaired Restriction[4] N (% of Total)	Diffusion Impaired Restriction Unadjusted OR [95% CI][1,2]	Diffusion Impaired Restriction Adjusted OR [95% CI][2,3]
>12 Months	50	22 (44)	0.56 [0.27-1.08]	0.58 [0.22-1.36]
ICU Admission				
No	217	101 (47)	—	—
Yes	121	90 (74)	3.35 [2.15-5.43]	1.20 [0.53-3.09]
COVID-19 Severity				
(4) Room Air	72	20 (28)	—	—
(5) Nasal Cannula	130	72 (55)	3.35 [1.80-6.30]	3.86 [1.79-9.17]
(6) High-Flow Cannula	83	54 (65)	5.04 [2.46-10.0]	4.72 [1.78-15.9]
(7) Ventilation	53	45 (85)	15.1 [6.47-46.8]	15.2 [4.72-68.1]

[1]Unadjusted Odds Ratio 95% confidence interval (n=1000 bootstraps)
[2]OR = Odds Ratio, CI = Confidence Interval
[3]Adjusted Odds Ratio 95% confidence interval (n=1000 bootstraps)
[4]Diffusion impaired restriction is defined by a DLCO ≤80% and a TLC ≤70% measured by PFT at the 1st Long COVID clinic visit

Supplemental Table 8: Outcome Modeling of Pulmonary Long COVID with Diffusion Impaired Restriction from CT Image Pathology

	Patients Total N	Diffusion Impaired Restriction[4] N (% of Total)	Diffusion Impaired Restriction Unadjusted OR [95% CI][1,2]	Diffusion Impaired Restriction Adjusted OR [95% CI][2,3]
Ground Glass Opacities				
No	116	54 (47)	—	—
Yes	192	150 (78)	4.11 [2.50-6.96]	1.40 [0.75-2.78]
Reticulations				
No	121	53 (44)	—	—
Yes	187	151 (81)	5.49 [3.24-9.35]	2.12 [1.01-4.34]
Other Fibrosis				
No	195	104 (53)	—	—
Yes	113	100 (88)	6.79 [3.59-15.0]	2.30 [0.95-5.96]
Bronchiectasis				
No	176	92 (52)	—	—
Yes	132	112 (85)	5.27 [3.10-9.60]	2.00 [0.92-4.82]
Consolidation				
No	277	179 (65)	—	—
Yes	31	25 (81)	2.35 [1.02-9.06]	1.39 [0.57-5.40]
Emphysema				
No	274	180 (66)	—	—
Yes	34	24 (71)	1.27 [0.60-3.15]	1.06 [0.42-2.99]

[1]Unadjusted Odds Ratio 95% confidence interval (n=1000 bootstraps)
[2]OR = Odds Ratio, CI = Confidence Interval
[3]Adjusted Odds Ratio 95% confidence interval (n=1000 bootstraps)
[4]Diffusion impaired restriction is defined by a DLCO ≤80% and a TLC ≤70% measured by PFT at the 1st Long COVID clinic visit

Supplemental Table 9: Characteristics of Pulmonary Long COVID Patients Stratified by Primary SARS-CoV-2 Infection Severity in the UAB Cohort

**Characteristic**	**WHO Score** N=929	**(3) No Admission** n=477	**(4) Room Air** n=105	**(5) Nasal Cannula** n=171	**(6) High-Flow Cannula** n=103	**(7) Mechanical Ventilation** n=73
Age[1]	53±14	50±14	54±14	56±13	60±12	55±11
Sex						
Female	609 (66)	331 (69)	83 (79)	107 (63)	53 (51)	35 (48)
Male	320 (34)	146 (31)	22 (21)	64 (37)	50 (49)	38 (52)
Race						
White	590 (64)	319 (67)	65 (62)	99 (58)	62 (60)	45 (62)
African American	261 (28)	117 (25)	35 (33)	58 (34)	31 (30)	20 (27)
Asian	21 (2)	10 (2)	2 (2)	4 (2)	2 (2)	3 (4)
Hispanic	14 (2)	5 (1)	0 (0)	4 (2)	3 (3)	2 (3)
American Indian	2 (0)	2 (0)	0 (0)	0 (0)	0 (0)	0 (0)
Multiple	2 (0)	0 (0)	1 (1)	0 (0)	1 (1)	0 (0)
Decline/Refuse	23 (2)	14 (3)	2 (2)	1 (1)	3 (3)	3 (4)
Not Reported	16 (2)	10 (2)	0 (0)	5 (3)	1 (1)	0 (0)
Body-mass Index[1]	33±9	32±8	34±8	34±9	35±9	34±9
Pre-COVID Comorbidities						
Pulmonary Disease	211 (23)	97 (20)	33 (31)	41 (24)	20 (19)	20 (27)
Renal Disease	66 (7)	12 (3)	9 (9)	17 (10)	16 (16)	12 (16)
Diabetes	168 (18)	60 (13)	25 (24)	44 (26)	24 (23)	15 (21)
Heart Failure or Hypertension	415 (45)	166 (35)	58 (55)	83 (49)	64 (62)	44 (60)
Obstructive Sleep Apnea	198 (21)	84 (18)	25 (24)	46 (27)	26 (25)	17 (23)
Smoking History						
Never smoker	637 (69)	334 (70)	73 (70)	121 (71)	58 (56)	51 (70)
Former smoker	249 (27)	122 (26)	26 (25)	42 (25)	40 (39)	19 (26)
Current smoker	43 (5)	21 (4)	6 (6)	8 (5)	5 (5)	3 (4)
Vaccination Status	184 (20)	118 (25)	25 (24)	20 (12)	10 (10)	11 (15)

Supplemental Table 9: Characteristics of Pulmonary Long COVID Patients Stratified by Primary SARS-CoV-2 Infection Severity in the UAB Cohort

**Characteristic**	**WHO Score** N=929	**(3) No Admission** n=477	**(4) Room Air** n=105	**(5) Nasal Cannula** n=171	**(6) High-Flow Cannula** n=103	**(7) Mechanical Ventilation** n=73
Primary COVID Infection						
SARS-CoV-2 Variant Wave						
Alpha Wave	583 (63)	302 (63)	65 (62)	114 (67)	62 (60)	40 (55)
Delta Wave	203 (22)	92 (19)	18 (17)	39 (23)	32 (31)	22 (30)
Omicron Wave	143 (15)	83 (17)	22 (21)	18 (11)	9 (9)	11 (15)
Immunosuppressed	155 (34)	0	9 (9)	89 (52)	29 (28)	28 (38)
Remdesivir Given	207 (38)	0 (0)	1 (1)	87 (56)	69 (68)	50 (69)
Dexamethasone Given	284 (52)	9 (8)	4 (4)	123 (78)	87 (84)	61 (85)
ICU Admission	161 (17)	0 (0)	0 (0)	13 (8)	77 (75)	71 (97)
Nasal Cannula Time (days)[2]	6 [4-10]	–	–	5 [3-7]	8 [7-11]	14 [7-21]
High-flow Nasal Cannula Time (days)	5 [3-9]	–	–	–	6 [3-9]	4 [2-6]
Ventilation Time (days)[2]	18 [8-34]	–	–	–	–	18 [8-34]
1st Long COVID Clinic Visit						
Primary Symptom						
Dyspnea	725 (78)	364 (76)	59 (56)	144 (84)	91 (88)	67 (92)
Cough	167 (18)	89 (19)	37 (35)	24 (14)	11 (11)	6 (8)
Chest Discomfort	37 (4)	24 (5)	9 (9)	3 (2)	1 (1)	0 (0)
Primary Infection to Long COVID Visit (days)[2]	131 [75-255]	141 [77-289]	121 [74-260]	122 [72-194]	116 [69-211]	130 [90-175]
1st Visit Lung Restriction						
Severe ≤50% TLC	129 (14)	23 (5)	8 (8)	29 (17)	29 (28)	40 (55)
Moderate 51-70% TLC	313 (34)	139 (29)	31 (30)	77 (45)	43 (42)	23 (32)
Mild 71-80% TLC	206 (22)	123 (26)	27 (26)	29 (17)	20 (19)	7 (10)
None >80% TLC	281 (30)	192 (40)	39 (37)	36 (21)	11 (11)	3 (4)
1st Visit Lung Diffusion						

Supplemental Table 9: Characteristics of Pulmonary Long COVID Patients Stratified by Primary SARS-CoV-2 Infection Severity in the UAB Cohort						
**Characteristic**	**WHO Score** N=929	**(3) No Admission** n=477	**(4) Room Air** n=105	**(5) Nasal Cannula** n=171	**(6) High-Flow Cannula** n=103	**(7) Mechanical Ventilation** n=73
Impaired ≤80% DLCO	574 (62)	217 (45)	60 (57)	139 (81)	88 (85)	70 (96)
Normal >80% DLCO	355 (38)	260 (55)	45 (43)	32 (19)	15 (15)	3 (4)
Follow-up Long COVID Clinic Visits TLC and DLCO[1]						
TLC (%) - 1st Visit	71±19	77±17	75±16	67±18	61±16	51±16
TLC, (%) - 2nd Visit	68±16	73±16	71±13	65±15	65±15	57±17
TLC, (%) - 3rd Visit	66±15	68±15	70±14	68±15	60±17	55±9
DLCO (%) - 1st Visit	73±23	82±19	77±18	65±21	58±19	46±18
DLCO, (%) - 2nd Visit	72±22	80±21	79±18	70±21	64±18	55±21
DLCO, (%) - 3rd Visit	69±20	78±17	75±16	70±21	63±19	53±16
CT Imaging						
CT Scan at 1st Long COVID Clinic Visit	308	108 (22)	20 (19)	76 (44)	57 (55)	47 (64)
Time from 1st Visit to CT Scan (days)[2]	28 [7-83]	25 [9-83]	38 [15-97]	30 [6-84]	24 [7-55]	34 [7-96]
CT Scoring Evaluations[2]						
CT Lung Involvement (0-25 Score)	6 [0-15]	0 [0-3]	2 [0-4]	8 [2-16]	13 [8-18]	19 [14-23]
Right Upper Lung (0-5)	1 [0-3]	0 [0-0]	0 [0-0]	1 [0-3]	3 [1-4]	4 [3-5]
Right Middle Lung (0-5)	1 [0-3]	0 [0-0]	0 [0-1]	1 [0-3]	3 [1-4]	4 [3-5]
Right Lower Lung (0-5)	1 [0-3]	0 [0-1]	0 [0-1]	2 [1-3]	3 [1-4]	4 [3-5]
Left Upper Lung (0-5)	1 [0-3]	0 [0-0]	0 [0-1]	1 [0-3]	2 [1-4]	4 [3-5]
Left Lower Lung (0-5)	1 [0-3]	0 [0-1]	0 [0-1]	2 [0-3]	2 [1-4]	4 [3-5]
CT Pathology						
Ground Glass Opacities	192 (62)	42 (39)	6 (30)	55 (72)	45 (79)	44 (94)
Reticulations	187 (61)	36 (33)	8 (40)	53 (70)	47 (82)	43 (91)

Supplemental Table 9: Characteristics of Pulmonary Long COVID Patients Stratified by Primary SARS-CoV-2 Infection Severity in the UAB Cohort

Characteristic	WHO Score N=929	(3) No Admission n=477	(4) Room Air n=105	(5) Nasal Cannula n=171	(6) High-Flow Cannula n=103	(7) Mechanical Ventilation n=73
Other Fibrosis	113 (37)	13 (12)	3 (15)	26 (34)	37 (65)	34 (72)
Bronchiectasis	132 (43)	22 (20)	5 (25)	38 (50)	39 (68)	28 (60)
Consolidation	31 (10)	5 (5)	1 (5)	7 (9)	6 (11)	12 (26)
Emphysema	34 (11)	7 (6)	3 (15)	5 (7)	8 (14)	11 (23)
Gas Trapping (Expiratory Only)	142 (67)	49 (69)	11 (92)	40 (73)	29 (69)	13 (42)

Statistics are reported as n, (%) unless otherwise specified by [1]Mean±SD or [2]Median [Q1-Q3]
N=total patients , n=total patients per WHO Score stratification group
Lung Involvement was assessed with a 0-5 scale (0=no involvement, 1=1-5%, 2=5-25%, 3=25-50%, 4=50-75%, 5≥75%)
Abbreviations: TLC, percent predicted total lung capacity; DLCO, diffusion limitation of carbon monoxide; CT, computerized tomography; WHO, World Health Organization; COVID, coronavirus disease; UAB, University of Alabama at Birmingham

Supplemental Table 10: Outcome Modeling of Pulmonary Long COVID with Diffusion Impaired Restriction (Sensitivity Testing with NICE Definition of Long COVID)

	Patients Total N	Diffusion Impaired Restriction[2] N (% of Total)	Diffusion Impaired Restriction Unadjusted OR [95% CI][1]	Diffusion Impaired Restriction Adjusted OR [95% CI][1]
Advanced Age				
<65 years	488	178 (36)	—	—
≥65 years	142	64 (45)	1.43 [0.98-2.05]	1.02 [0.64-1.64]
Sex				
Female	412	140 (34)	—	—
Male	218	102 (47)	1.72 [1.21-2.37]	1.44 [0.96-2.09]
Elevated BMI				
<30	253	95 (38)	—	—
≥30	377	147 (39)	1.07 [0.76-1.48]	1.00 [0.66-1.56]
Pulmonary Disease				
No	492	186 (38)	—	—
Yes	138	56 (41)	1.12 [0.75-1.62]	1.01 [0.63-1.56]
Renal Disease				
No	590	220 (37)	—	—
Yes	40	22 (55)	2.09 [1.11-4.38]	0.82 [0.36-1.91]
Diabetes				
No	512	185 (36)	—	—
Yes	118	57 (48)	1.64 [1.09-2.46]	1.07 [0.60-1.84]
Heart Failure or Hypertension				
No	352	103 (29)	—	—
Yes	278	139 (50)	2.44 [1.73-3.41]	2.14 [1.38-3.24]
Obstructive Sleep Apnea				
No	501	196 (39)	—	—
Yes	129	46 (36)	0.85 [0.57-1.27]	0.56 [0.33-0.89]
Smoking History				
Never smoker	440	162 (37)	—	—
Current or Former Smoker	190	80 (42)	1.25 [0.86-1.76]	1.10 [0.72-1.65]
Vaccination Status				
No	531	203 (38)	—	—
Yes	99	39 (39)	1.06 [0.67-1.61]	1.33 [0.79-2.30]
Months from Primary Infection to Long COVID Clinic Visit				
3-6 Months	296	141 (48)	—	—

Supplemental Table 10: Outcome Modeling of Pulmonary Long COVID with Diffusion Impaired Restriction (Sensitivity Testing with NICE Definition of Long COVID)

	Patients Total N	Diffusion Impaired Restriction[2] N (% of Total)	Diffusion Impaired Restriction Unadjusted OR [95% CI][1]	Diffusion Impaired Restriction Adjusted OR [95% CI][1]
6-12 Months	200	59 (30)	0.46 [0.32-0.65]	0.72 [0.47-1.10]
≥12 Months	134	42 (31)	0.50 [0.32-0.75]	0.67 [0.38-1.11]
ICU Admission				
No	519	162 (31)	—	—
Yes	111	80 (72)	5.70 [3.72-9.26]	0.89 [0.31-2.75]
COVID-19 Severity				
(3) No Admission	330	75 (23)	—	—
(4) Room Air	68	19 (28)	1.33 [0.71-2.27]	1.26 [0.63-2.46]
(5) Nasal Cannula	110	61 (55)	4.21 [2.72-6.75]	4.20 [2.53-7.02]
(6) High-Flow Cannula	67	41 (61)	5.38 [2.98-10.2]	5.62 [1.82-16.2]
(7) Ventilation	55	46 (84)	17.9 [8.43-51.6]	19.1 [5.34-72.8]

[1]Unadjusted odds ratio (OR) and adjusted odds ratio (aOR), 95% confidence interval (n=1000 bootstraps)
[2]Diffusion impaired restriction is defined by a DLCO ≤80% and a TLC ≤70% measured by PFT at the 1st Long COVID clinic visit
NICE Definition of Long COVID excluded patients with Long COVID clinic visits within 90-days of their Primary COVID Infection

Supplemental Table 11: Pulmonary Function Test and CT Image Comparison for Pulmonary Long COVID Patients with 3-Followup Visits (n=17 patients)

	**UAB Pulmonary Long COVID Clinic Visit**		
	1st Visit n=17	2nd Visit n=17	3rd Visit n=17
Pulmonary Function Tests[1]			
TLC (% predicted)	54 [43-68]	60 [52-65]	62 [54-66]
DLCO (% predicted)	66 [39-73]	64 [45-75]	70 [50-74]
CT Scoring[1]			
CT Score Overall (0-25)	10 [5-17]	11 [4-19]	10 [4-20]
CT Score Right Upper Lung (0-5)	2 [1-4]	2 [1-4]	2 [1-4]
CT Score Right Middle Lung (0-5)	2 [1-4]	2 [0-4]	2 [1-4]
CT Score Right Lower Lung (0-5)	2 [1-4]	3 [2-4]	2 [1-4]
CT Score Left Upper Lung (0-5)	2 [1-3]	2 [0-4]	2 [1-4]
CT Score Left Lower Lung (0-5)	2 [1-4]	2 [2-4]	2 [1-4]
CT Pathology			
Bronchiectasis	9 (53)	8 (47)	9 (53)
Consolidation	1 (6)	2 (12)	3 (18)
Emphysema	1 (6)	1 (6)	1 (6)
Ground Glass Opacities	13 (76)	15 (88)	14 (82)
Fibrotic Changes	8 (47)	7 (41)	6 (35)
Reticulations	13 (76)	14 (82)	14 (82)

Statistics are reported as n, (%) unless otherwise specified by [1]Median [Q1-Q3]
Lung Involvement was assessed with a 0-5 scale (0=no involvement, 1=1-5%, 2=5-25%, 3=25-50%, 4=50-75%, 5≥75%)
